# Supplementary material for: CMOS backend-of-line compatible memory array and logic circuitries enabled by high performance atomic layer deposited ZnO thin-film transistor
Source: Nat Commun. 2023 Sep 28;14:6079. doi: 10.1038/s41467-023-41868-5 (PMC10539278; doi:10.1038/s41467-023-41868-5)
Supplement: Supplementary file 1 — Supplementary Information [file 41467_2023_41868_MOESM1_ESM.pdf]

Supplementary Information

CMOS Backend-of-Line Compatible Memory Array and Logic Circuitries Enabled by High Performance Atomic Layer Deposited ZnO Thin-film Transistor

Wenhui Wang[1=], Ke Li[1=], Jun Lan[1], Mei Shen[1], Zhongrui Wang[2], Xuewei Feng[3], Hongyu Yu[1], Kai Chen[1], Jiamin Li[1], Feichi Zhou[1], Longyang Lin[1*], Panpan Zhang[4*], and Yida Li[1*]

[1]School of Microelectronics, Southern University of Science and Technology, Shenzhen, China 518055

[2]Department of Electrical and Electronic Engineering, The University of Hong Kong, Hong Kong SAR 999077, China

[3]Shanghai Jiao Tong University, Shanghai, China 200240

[4]State Key Laboratory of Information Photonics and Optical Communications, Beijing University of Posts and Telecommunications, Beijing 100876, China

[=]equal contributions; [*]linly@sustech.edu.cn, tanji_ic@bupt.edu.cn, liyd3@sustech.edu.cn

Supplementary Figures

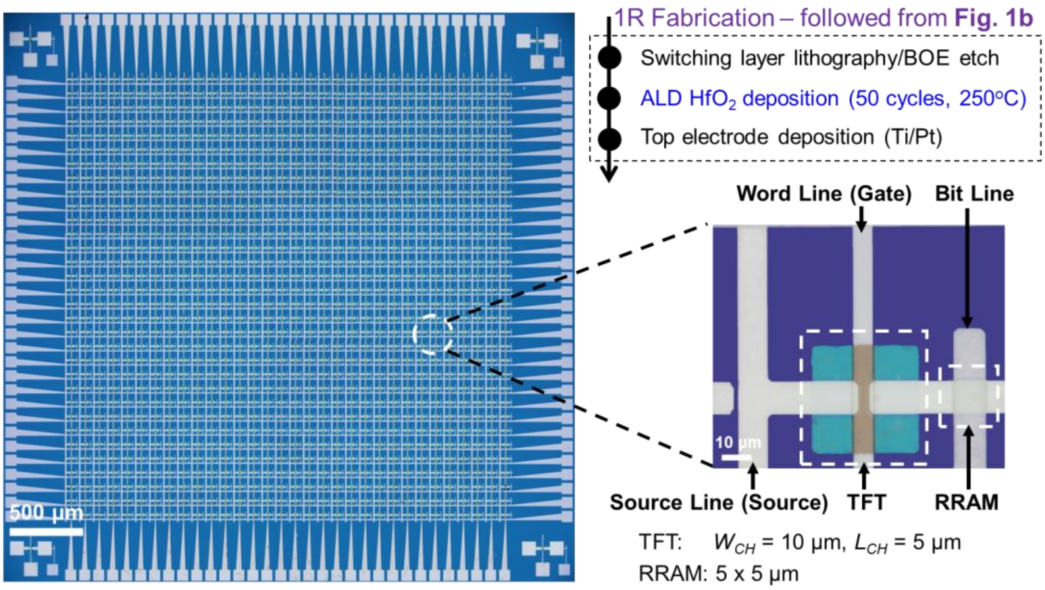

Supplementary Figure S1 | 1T1R array. Process integration flow of the RRAM in the 1T1R array, microscopic image of the fabricated 1 kbit (32×32) 1T1R array, and a zoom-in image of one single 1T1R cell. The TFT channel width and length used in 1T1R array are 10 μm and 5 μm, respectively. The size of 5nm thick HfO_2-based RRAM is 5 μm × 5μm.

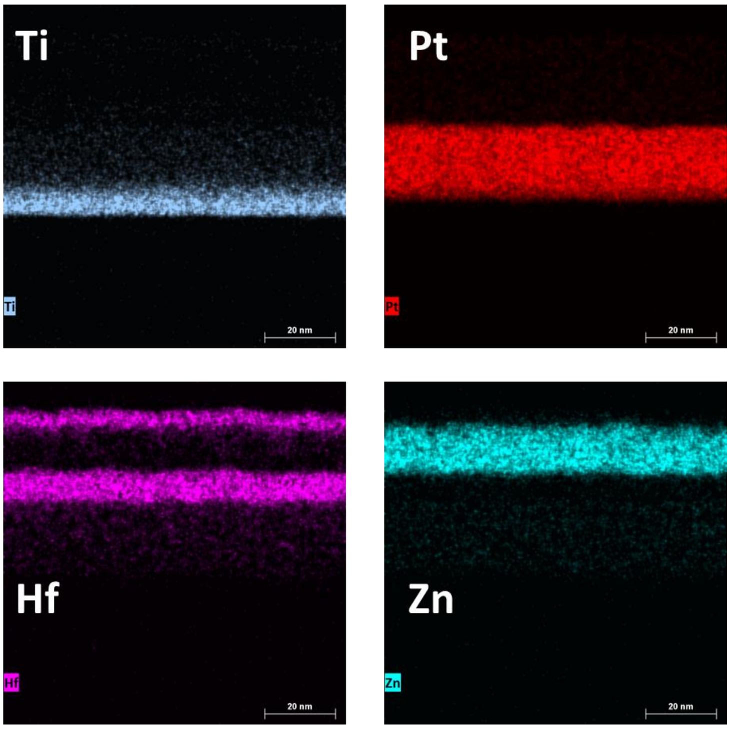

Supplementary Figure S2 | EDS mapping of the ZnO TFT stack. The EDS mapping of Ti, Pt, Hf, and Zn elements taken from the channel material stack shows clear distinction of the different layers.

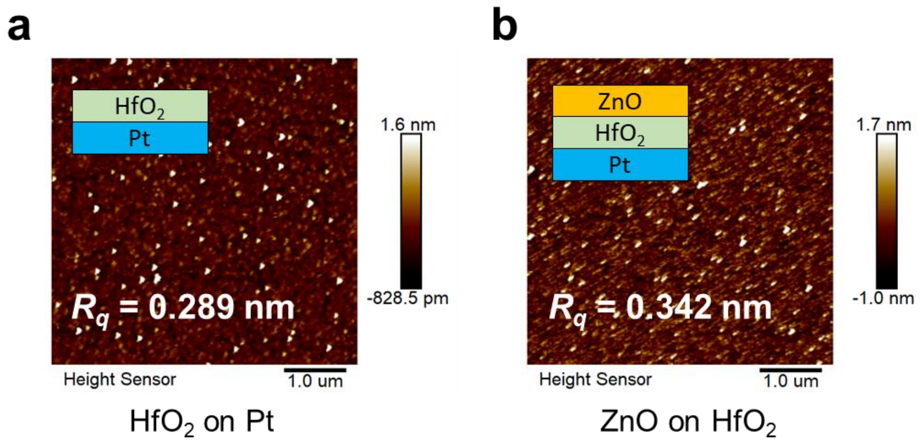

Supplementary Figure S3 | AFM images. a, b AFM images of the HfO_2 dielectric on gate electrode, and ZnO on HfO_2 dielectric on gate during fabrication. The average R_q of HfO_2 on gate and ZnO on HfO_2 dielectric on gate is 0.289 nm (standard deviation: 0.006 nm, over 3 independent scans) and 0.342 nm (standard deviation: 0.013 nm, over 3 independent scans), respectively.

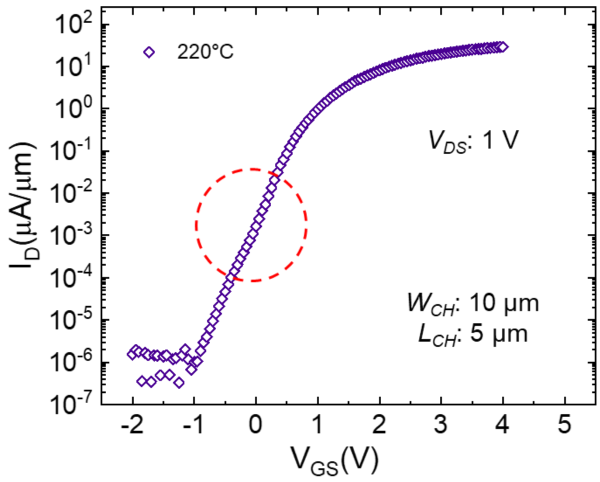

Supplementary Figure S4 | Transfer curve of 220°C deposited ZnO TFT. The transfer curve of 220°C deposited ZnO TFT, with an observable small hump in the subthreshold region (circled dashed line).

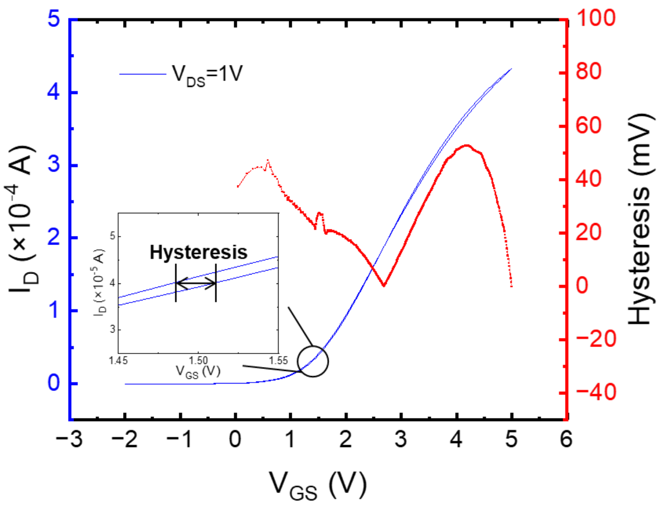

Supplementary Figure S5 | Hysteresis Extraction. Extracted hysteresis from Fig. 3c as a function of V_{GS} at a V_{DS} of 1 V, with a maximum hysteresis value of 52 mV. The definition of the hysteresis extraction is shown graphically in the inset.

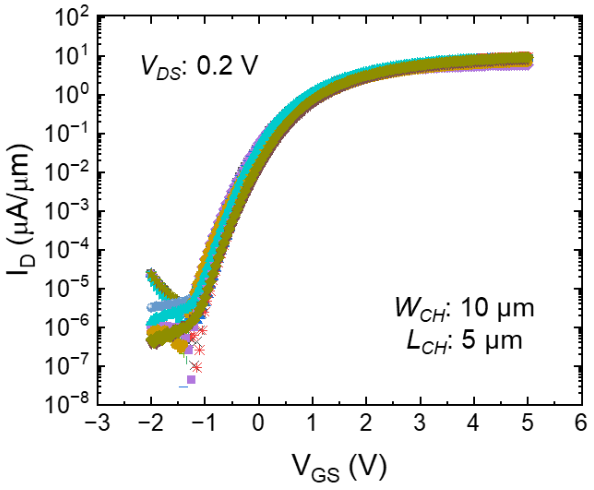

Supplementary Figure S6 | ZnO TFT uniformity. Transfer curves of 20 different ZnO TFTs measured randomly. Similar performance of the devices was verified with minor variations.

Parameter	Mean value	Standard deviation
V_{TH} (V)	0.81	0.09
μ_{FE} (cm^2/V·s)	75.89	7.37
SS (mV/dec)	132.8	13.1
I_{on}/I_{off}	1.47×10^{8}	4.4×10^{7}
D_{it} ($eV^{-1}cm^{-2}$)	3.34×10^{11}	5.8×10^{10}

The mean value and standard deviation of the extracted parameters in Supplementary Figure S6

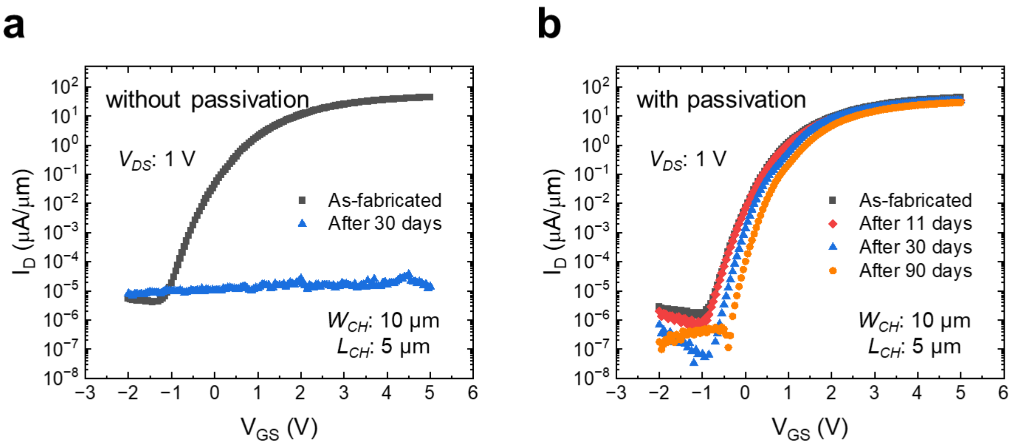

Supplementary Figure S7 | Passivation effect on performance of ZnO TFT. a Evolution of transfer curves of the un-passivated ZnO TFT over time with significant degradation. b Evolution of transfer curves of the passivated ZnO TFT over time with minor change.

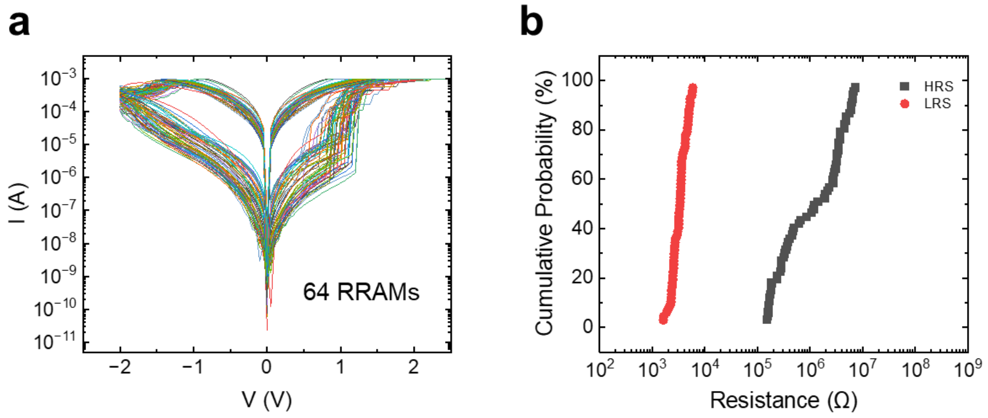

Supplementary Figure S8 | Multiple RRAMs DC characteristics. a DC characteristic of 64 HfO_2 RRAMs, b Cumulative probability plot of HRS and LRS distribution for the 64 RRAMs.

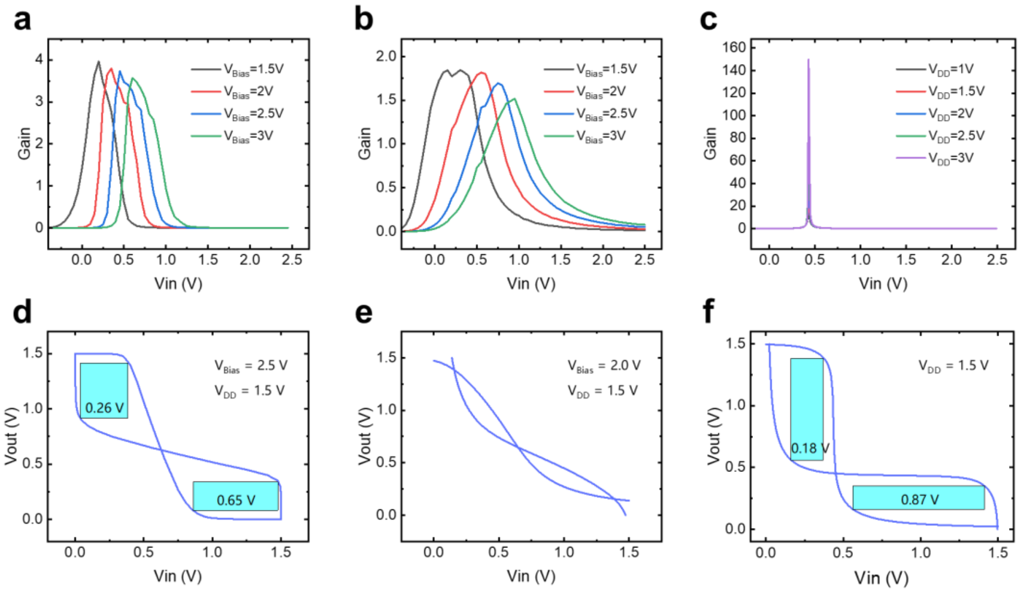

Supplementary Figure S9 | Gain and Noise Margin (NM) of PEL, LEL, and DL inverters. a, b Gain of PEL and LEL inverters, with V_{DD} of 1.5 V and V_{Bias} varying from 1.5 V to 3 V. c Gain of DL inverter with V_{DD} varying from 1 V to 3V. d, e, f NM of PEL, LEL and DL inverters with V_{DD} of 1.5 V. The NM of LEL inverter is not ideal and near-zero.

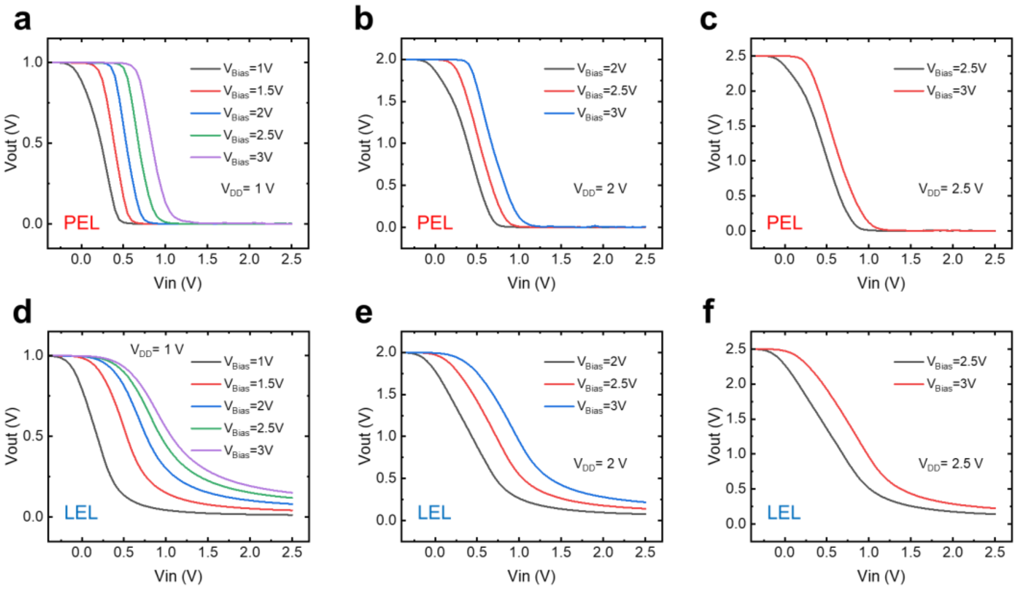

Supplementary Figure S10 | VTCs of inverters under different V_{DD}. a, b, c VTCs of PEL inverter under V_{DD} of 1 V, 2 V, and 2.5 V, respectively. d, e, f VTCs of LEL inverter under V_{DD} of 1 V, 2 V, and 2.5 V, respectively.

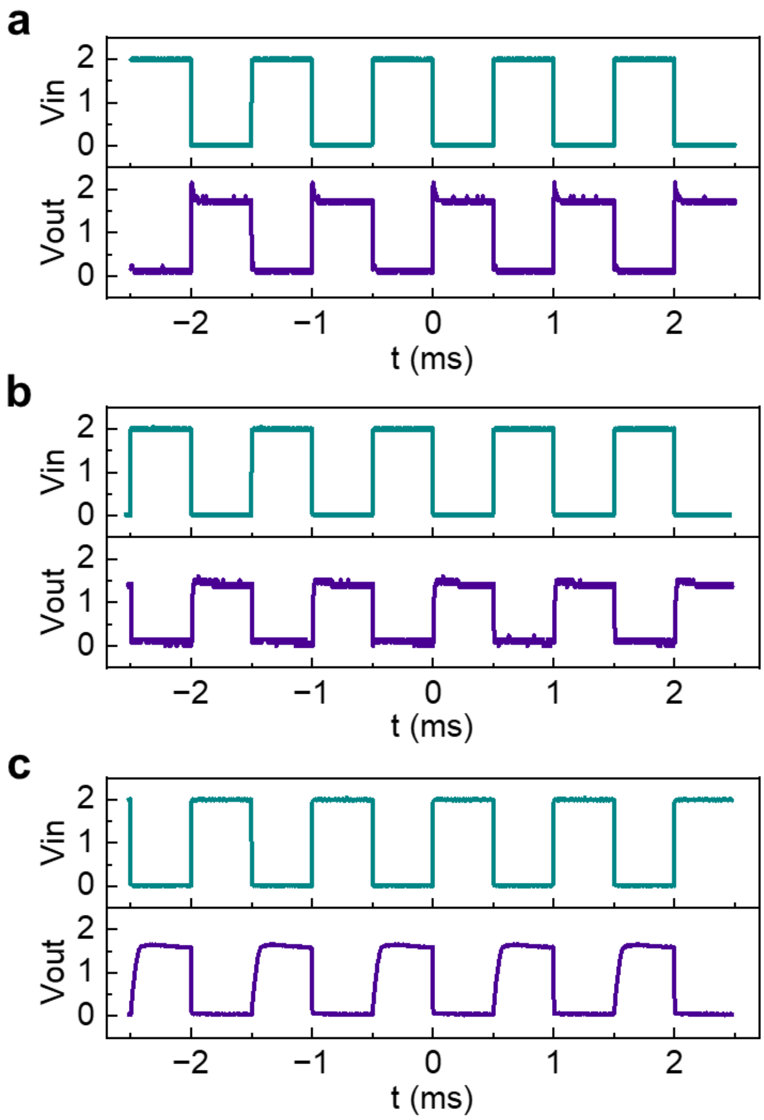

Supplementary Figure S11 | Square pulse responses of the fabricated inverters. Square pulse responses for a PEL, b LEL, and c DL inverter with input frequency of 1 kHz. The V_{Bias} and V_{DD} for inverter are both 2 V.

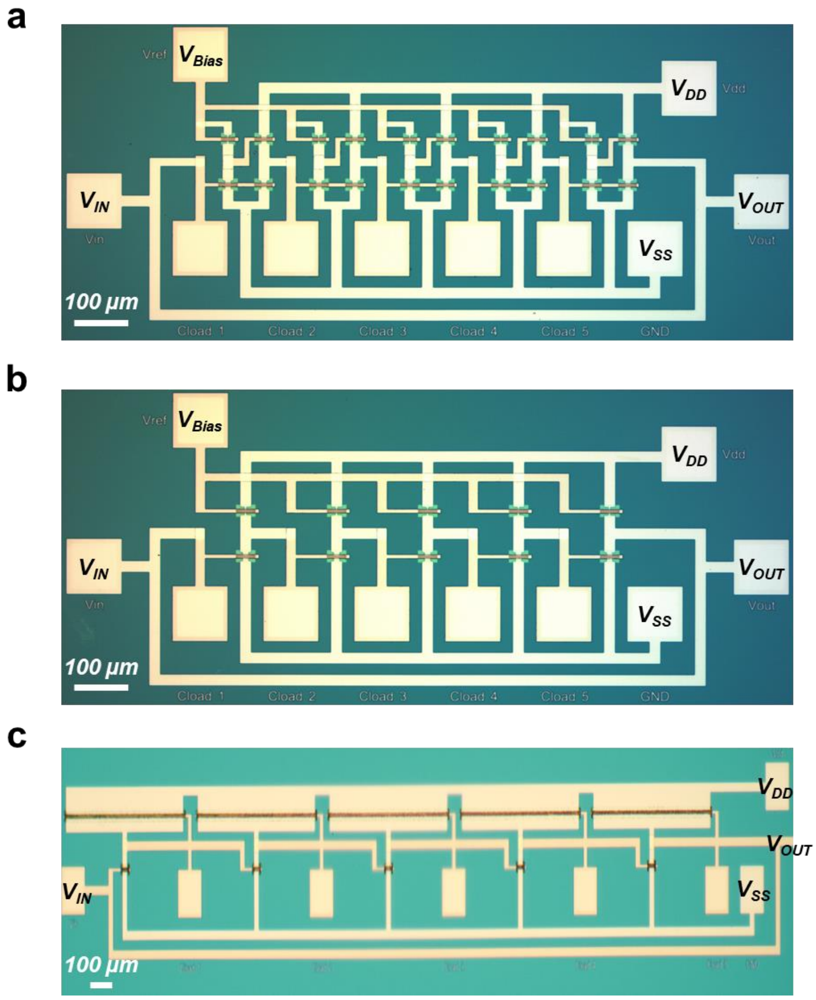

Supplementary Figure S12 | Optical images of ring oscillators. a PEL inverter-based ring oscillator image, b LEL inverter-based ring oscillator image, **c** DL inverter-based ring oscillator image.

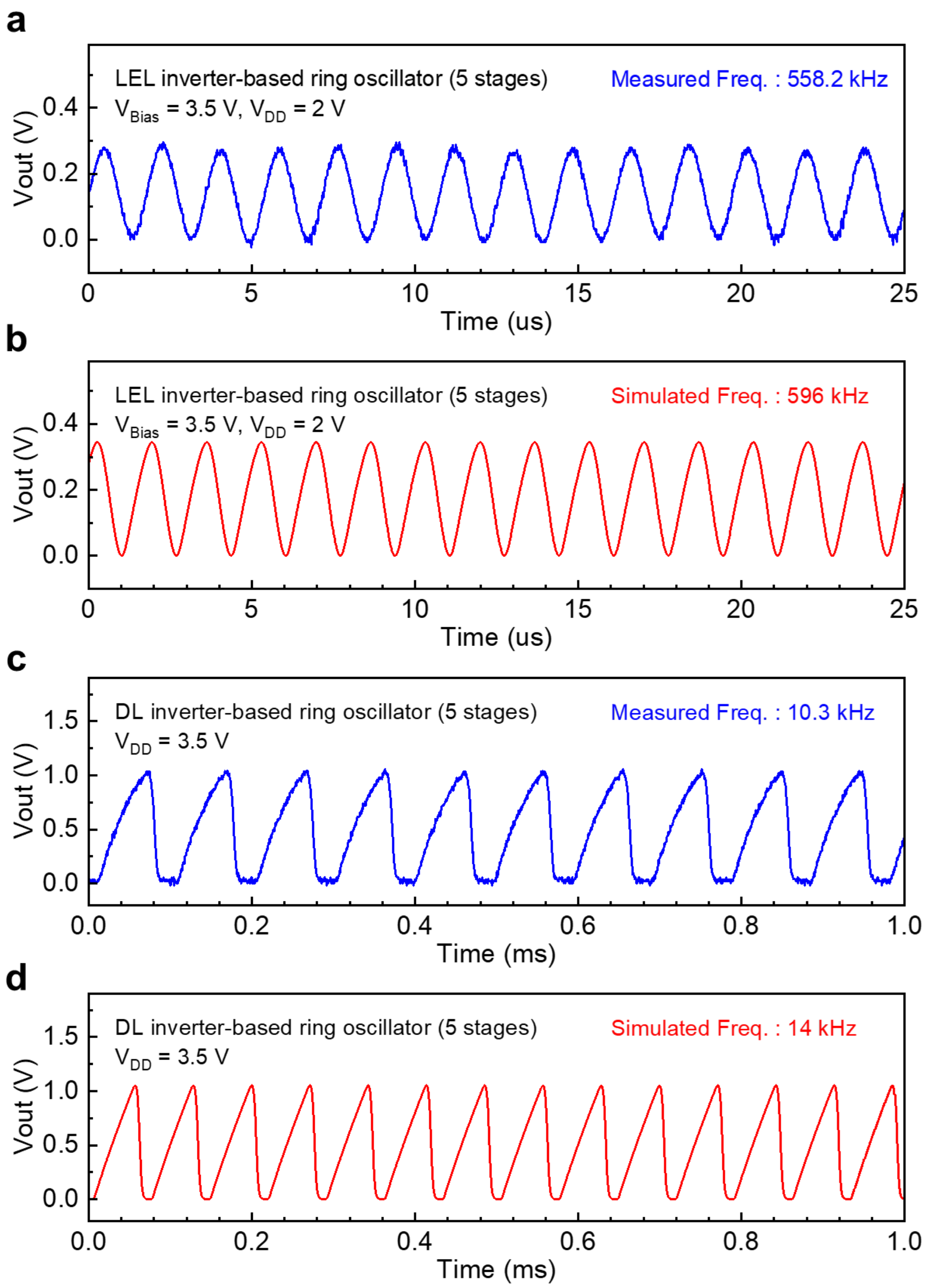

Supplementary Figure 13 | Ring Oscillator output waveforms. a, b The as-measured and simulated output waveforms for the 5-stage LEL inverter-based ring oscillator with frequency of 558.2 kHz and 596 kHz, respectively. c, d The as-measured and simulated output waveforms for the 5-stage DL inverter-based ring oscillator with frequency of 10.3 kHz and 14 kHz, respectively.

Supplementary Tables

No.	Equation	Remark
**1**	$I_{DS}=\frac{I_{sub}+I_{above}}{I_{sub}*I_{above}}$	I_{sub}: subthreshold current<br>I_{above}: above threshold current
**2**	$I_{above}=\mu_{FET}C_{ox}\frac{W}{L}[(V_{GS}-V_T)-\frac{V_{DSE}}{2\alpha}]V_{DSE}$	V_{DSE} : effective drain-source voltage
**3**	$1/\mu_{FET}=1/\mu_0+1/\mu_1\cdot\left\|\frac{2(V_{GS}-V_T)}{\eta V_{th}}\right\|^m$	m: field enhancement factor for the mobility<br>μ_0: low-field mobility
**4**	$V_{DSE}=\frac{V_{DS}}{[1+(\frac{V_{DS}}{V_{DSat}})^\beta]^{1/\beta}}$	β: fitting parameter of smooth function
**5**	$I_{sub}=\mu_s C_{ox}\frac{W}{L}(\eta V_{th})^2\exp\left(\frac{V_{GS}-V_T}{\eta V_{th}}\right)\cdot[1-\exp(\frac{-V_{DS}}{\eta V_{th}})]$	$V_{th}=\kappa T/q$: thermal voltage
**6**	$SS\approx\frac{kT}{q}ln10\cdot(1+qD_{it}/C_{ox})$	D_{it}: density of states of interface traps

Supplementary Table S1 | Main equations to implement the model.

Inverter type	Noise Margin NM_H/ NM_L (V)	Gain	Rail to rail
PEL	0.65/ 0.26	4.0	Yes
LEL	/	1.84	No
DL	0.87/ 0.18	149.9	Yes

Supplementary Table S2 | Benchmark of the three fabricated inverters performance for different configurations. The NM of LEL inverter is not ideal and near-zero.

V_{Bias}/V_{DD} (V)	***Freq.*** **(kHz)**	τ_p **(μS)**	***Vpp*** **(V)**	I_{DD} **(μA)**
2/2	93.7	1.07	1.04	12.0
2.5/2	158.1	0.63	1.18	23.5
3/2	251.8	0.40	1.18	41.8
3.5/2	369.1	0.27	1.04	66.2
2.5/2.5	155.0	0.65	1.18	23.7
3/2.5	241.6	0.41	1.38	42.8
3.5/2.5	347.1	0.29	1.46	68.4
3/3	241.4	0.41	1.44	44.5
3.5/3	340.0	0.29	1.62	69.9

Supplementary Table S3 | PEL inverter-based RO performance parameters. The measured frequency, peak-to-peak voltage, working current, and calculated delay time per stage under different V_{Bias}/V_{DD} pairs.

V_{Bias}/V_{DD} (V)	*Freq.* (kHz)	τ_p (μS)	V_{pp} (V)	I_{DD} (μA)
2/2	150.9	0.66	0.43	36.0
2.5/2	258.2	0.39	0.46	66.5
3/2	391.7	0.26	0.31	103.0
3.5/2	558.3	0.18	0.32	162.4
2.5/2.5	251.2	0.40	0.57	78.8
3/2.5	374.2	0.27	0.62	129.4
3.5/2.5	523.8	0.19	0.57	179.9
3/3	370.9	0.27	0.71	140.6
3.5/3	504.6	0.20	0.74	211.4

Supplementary Table S4 | LEL inverter-based RO performance parameters. The measured frequency, peak-to-peak voltage, working current, and calculated delay time per stage under different V_{Bias}/V_{DD} pairs.

V_{DD} **(V)**	***Freq.*** **(kHz)**	***τ_p*** **(µS)**	***V_{pp}*** **(V)**	***I_{DD}*** **(µA)**
1.5	8.4	11.9	0.88	4.16
2	8.8	11.4	0.96	4.71
2.5	9.6	10.4	1.02	5.51
3	10.0	10.0	1.06	5.87
3.5	10.3	9.7	1.10	6.20

Supplementary Table S5 | DL inverter-based RO performance parameters. The measured frequency, peak-to-peak voltage, working current, and calculated delay time per stage under different V_{DD} supply.

Supplementary Note

Supplementary Note S1 | Crystallite size calculation through Scherrer Equation

The average crystallite sizes of polycrystalline ZnO thin films under different process temperatures are calculated through Scherrer Equation, shown as,

$$D = \frac{K\gamma}{Bcos\theta}$$

where D is the mean size of the ordered crystalline domains, K is a dimensionless shape factor with a value close to unity (K = 0.89), γ is the X-ray wavelength (1.54056 Å), B is the line broadening at half the maximum intensity (FWHM) after subtracting the instrumental line broadening, in radians, θ is the Bragg angle.

The average crystallite sizes of the 3 different temperatures deposited ZnO films are 4.58 nm, 5.14 nm and 4.85nm, respectively. The separate B and θ value of each XRD spectra peak are shown in following table.

**150°C deposited ZnO film**		**200°C deposited ZnO film**		**250°C deposited ZnO film**	
**2θ (°)**	**FWHM (°)**	**2θ (°)**	**FWHM (°)**	**2θ (°)**	**FWHM (°)**
32.04	1.44	32.12	1.83	34.49	1.33
34.45	1.37	34.49	1.39	36.31	1.41
36.24	1.88	36.29	1.66	47.54	1.81
47.61	2.16	47.55	2.09	52.43	1.70
52.40	1.69	52.67	1.86	54.29	1.83
54.45	2.13	54.71	0.77	55.77	2.93
56.45	2.40	55.58	3.32	62.88	2.12
62.89	2.15	62.85	2.26	68.13	2.08
68.23	2.47	68.09	2.46		

Supplementary Note S2 | Mobility extraction method for TFT

The field-effect mobility (μ_{FE}) and intrinsic mobility (μ_o) are calculated using Eq (1) and (2),

$$\mu_{FE} = \frac{G_m}{C_{ox}\frac{W}{L}V_{DS}} \quad (1)$$

$$\mu_o = \frac{\mu_{FE}}{1-\frac{2R_{contact}}{R_{total}}} \quad (2)$$

,

where G_m is the transconductance extracted from transfer curves of TFT, W and L are the channel width (10μm) and length (5μm), V_{DS} is the applied drain voltage, C_{ox} is calculated from an extracted dielectric constant of ~15 using a metal-insulator-mental (MIM) capacitor, and R_{total} *and* $R_{contact}$ are acquired from Fig. 3b in main text.
